# Tamoxifen-induced ovarian hyperstimulation during premenopausal hormonal therapy for breast cancer in Japanese women

**DOI:** 10.1186/s40064-015-1223-0

**Published:** 2015-08-19

**Authors:** Rena Yamazaki, Masafumi Inokuchi, Satoko Ishikawa, Subaru Myojo, Junpei Iwadare, Yukiko Bono, Yasunari Mizumoto, Mitsuhiro Nakamura, Masahiro Takakura, Takashi Iizuka, Tetsuo Ohta, Hiroshi Fujiwara

**Affiliations:** Department of Obstetrics and Gynecology, Kanazawa University Graduate School of Medical Science, Kanazawa, Ishikawa 920-8641 Japan; Division of Cancer Medicine, Department of Breast Oncology, Kanazawa University Graduate School of Medical Science, 13-1 Takaramachi, Kanazawa, Ishikawa 920-8641 Japan

**Keywords:** Breast cancer, Estradiol, Gonadotropin, Hypothalamic-pituitary-axis, Ovarian hyperstimulation, Tamoxifen

## Abstract

**Purpose:**

Tamoxifen is an anti-estrogenic drug that is widely used for endocrine-dependent breast cancer as adjuvant hormonal therapy, and its use has been reported to be frequently associated with high levels of serum estradiol. Since the population of premenopausal women receiving tamoxifen therapy is growing in Japan, we retrospectively analyzed the incidence of ovarian hyperstimulation by tamoxifen therapy in Japanese women.

**Methods:**

Eleven patients who received surgical therapy for endocrine-dependent breast cancer and showed high values of serum estradiol during post-operative tamoxifen therapy were recruited in this study and evaluated by examining the serum concentration of follicular stimulating hormone (FSH) and follicular development.

**Results:**

The mean age, serum concentrations of estradiol and FSH, and follicular diameter were 41.3 years old, 1015.8 pg/mL, 11.8 mIU/mL, and 3.47 cm, respectively. In 6 cases, multiple follicular development was observed, while the other cases showed single follicular development with a mean serum estradiol level of 848.6 pg/mL and follicular diameter of 4.46 cm. There was no significant difference in age or FSH concentration between the two groups. The mean periods from the start of the single administration of tamoxifen to the initial detection of a high estradiol concentration was 716.5 days.

**Conclusions:**

These findings indicate that tamoxifen could stimulate the ovarian function even after 2-year treatment. Since single and multiple follicular developments with large sizes were observed, dual mechanisms through the inhibition of both negative and positive feedback to the hypothalamic-pituitary-axis can be proposed to explain the adverse effects of tamoxifen on ovarian function.

## Background

In contrast to other industrial countries, the incidence of breast cancers has constantly increased since the 1990s in Japan along with the habitual changes of food intake from Japanese to Western styles (Katanoda et al. 2015). Consequently, the Japanese population receiving hormonal therapy for estrogen-dependent breast cancer during the premenopausal period has also been gradually growing, with a concomitant increase in the incidence rate of breast cancer (Uchida et al. 2015).

Tamoxifen (TAM), a nonsteroidal anti-estrogen drug, is widely used for the treatment of patients with all stages of estrogen-dependent breast cancer, and the long-term single use of tamoxifen has been applied to treat patients during the premenstrual period (Burstein et al. 2014). In accordance with this trend, the Japanese Breast Cancer Society also recommended a single use of TAM more than for 5 years for premenstrual patients with estrogen-dependent breast cancer (Mukai et al. 2015).

On the other hand, it was reported that treatment with TAM as a single agent for estrogen-dependent breast cancer during the premenopausal period is associated with a one- to three-fold increase of the serum levels of estradiol and progesterone (Jordan et al. 1994). It was also proposed that TAM potentially stimulates the ovarian function accompanied by formation of persistent follicular functional cysts in the premenopausal women (Shulman et al. 1994; Shushan et al. 1996; Mourits et al. 1999; Cohen et al. 1999). Recently, Madeddu et al. (2014) reported two cases of multiple follicular development along with high estradiol concentrations of 1200 and 698.8 pg/mL, respectively and pointed out the possible involvement of the direct effects of tamoxifen on ovarian function (Groom and Griffiths 1976).

The pathological conditions of polycystic ovarian syndrome in Japan, which is associated with a disorder of hormonal conversion from androgen to estrogen in the growing follicles, were reported to be markedly different from those of Western countries (Mori et al. 2009). Therefore, we should note the possibility that the ovarian response of Japanese women against anti-estrogen drugs varies from that of women in Western countries. However, the side effects of TAM on the ovarian function have not yet been thoroughly analyzed in Japan. Therefore, in this study, we retrospectively analyzed Japanese patients who showed high levels of serum estradiol during TAM therapy for estrogen-dependent breast cancer during the premenopausal period.

## Methods

From August 2013 to August 2014, 62 patients who received post-operative TAM therapy for endocrine-dependent breast cancer (stage I–III) visited the out-patient clinic of the Department of Breast Oncology in Kanazawa University Hospital. When the serum concentration of estradiol was higher than 400 pg/mL, which exceeds the normal estradiol production by a single preovulatory follicle, these patients were immediately recommended to consult the Department of Gynecology in Kanazawa University Hospital. At the gynecological clinic, hormonal conditions were evaluated again by measuring serum concentrations of FSH and estradiol, while follicular development was further observed by ultrasonographic examination.

Among 62 candidates, 11 patients showed high values of serum estradiol during TAM therapy and visited the Department of Gynecology within 2 weeks following the previous blood sampling. These 11 cases were recruited into this study and hormonal and ultrasonographic data were retrospectively analyzed.

Estradiol and FSH were measured by electro-chemiluminescence immunoassay (ECLIA) kits (Roche Diagnostics K.K., Tokyo, Japan). Data on serum concentrations of estradiol and FSH and follicular diameters are expressed as means ± standard deviations and the differences between the single and multiple follicular development groups, and the chemotherapy-treated and untreated groups, were analyzed by the unpaired *t* test. On the other hand, the differences in durations of single TAM treatment until the initial detection of a high concentration of estradiol were analyzed by the Mann–Whitney *U*-test.

This study was approved by the Medical Ethics Committee of Kanazawa University.

## Results

The profiles of the 11 patients are summarized in Table 1. The mean age was 41.3 ± 7.34 years old. The serum concentrations of estradiol and FSH were 1015.8 ± 365.5 pg/mL and 11.8 ± 8.36 mIU/mL, respectively. The mean follicular diameter was 3.47 ± 1.91 cm. In 6 cases, multiple follicles (2–4 follicles) were observed and the mean serum concentration of estradiol and its value per follicle were 1155.2 ± 414.8 and 460.7 ± 195.8 pg/mL, respectively. On the other hand, the other 5 cases showed single follicular development and the mean serum concentration of estradiol and follicular diameter were 848.6 ± 234.2 pg/mL and 4.46 ± 2.33 cm, respectively. There was no significant difference in the age or FSH concentration between the two groups.

**Table 1:** Clinical profiles of 11 cases with high concentration of serum estradiol during TAM treatment

Case	Age	Stage	Chemo	E2 (pg/mL)	FSH (mIU/mL)	Ns of follicles	Estradiol/Ns (pg/mL)	Av of Fl dia. (cm)	Duration of TAM Tx (days)	Status of mensus within 3 months
1	54	I		1556	4.4	2	778	2	364	Regular
2	46	I		1028	9.3	2	514	4.7	872	Regular
3	29	IIa	Done	1600	9.9	4	400	2.5	889	Chemo-M
4	43	I		1202	8	4	300	2.5	1054	Regular
5	46	IIb	Done	464	24.9	2	232	1.5	427	Chemo-M
6	30	IIIa	Done	1081	12	2	540	2.7	137	Chemo-M
7	45	I		900	6.9	1	900	8.1	724	Regular
8	44	I		751	6	1	751	2.2	410	Irregular
9	36	IIa	Done	898	7.7	1	898	4.3	791	Irregular
10	40	IIa	Done	527	31.0	1	527	2.7	1825	Irregular
11	41	IIIc	Done	1167	9.5	1	1,167	5	389	Chemo-M

*NAC* neoadjuvant chemotherapy, *Ns* numbers, *dia.* diameters, *E2* estradiol, *Tx* treatment, *Fl* follicular, *Chemo* chemotherapy, *Chemo-M* chemotherapy-induced menopause

The mean duration from the start of the single administration of TAM to the initial detection of a high concentration of estradiol was 716.5 ± 463.4 days. In the group treated with chemotherapy, the mean duration was 743.0 ± 597.8 (137–1825) days, while the mean in the group without chemotherapy was 684.8 ± 296.4 (364–1054) days, showing no significant differences. Similarly, there were no differences in the estradiol concentration between the chemotherapy-treated group (956.7 ± 425.3 pg/mL) and non-treated group (1087.4 ± 310.1 pg/mL).

## Discussion

In this study, adverse effects of TAM on the ovarian function during hormone therapy for breast cancers were observed in Japanese women of reproductive age. Although the precise incidence rate is unclear, considering the low frequency of post-operative follow-up, 2 or 3 visits a year, the association of a high concentration of serum estradiol with TAM therapy may be a relatively common phenomenon in Japanese patients.

**Fig. 1:**
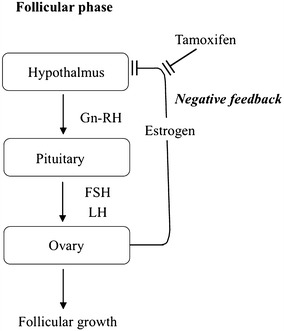
Inhibitory action of TAM on negative feedback to the hypothalamic-pituitary-axis by estrogen during the follicular phase. TAM can inhibit negative feedback to the hypothalamic-pituitary-axis by estrogen, leading to the increases of FSH and LH secretion by the pituitary gland and then inducing multiple follicular development

The formation of a couple of ovarian follicular cysts was observed not only in three cases with regular menstruation, but also in the other three cases that had been clinically diagnosed as chemotherapy-induced menopause. This suggests that TAM can induce multiple follicular development, which is usually observed in ovulation induction using an anti-estrogenic agent, clomiphene (Nasseri and Ledger 2001). Anti-estrogenic drugs are considered to inhibit the hypothalamic-pituitary-axis by negative feedback to stimulate the secretion of pituitary gonadotropins such as FSH and luteinizing hormone (LH) (Fig. 1). Since the pulsatile secretion of pituitary gonadotropins is important in the selection and/or promotion of follicular development (Skorupskaite et al. 2014), more examinations including the rhythmic changes in pulsatile secretion by TAM are necessary to clarify the precise mechanisms.

**Fig. 2:**
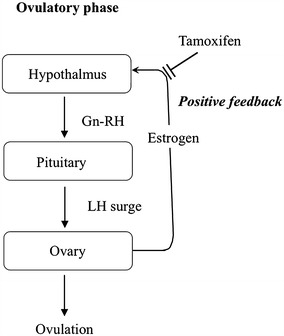
Inhibitory action of TAM on positive feedback to the hypothalamic-pituitary-axis by estrogen during the ovulatory phase. TAM can inhibit positive feedback to the hypothalamic-pituitary-axis by estrogen, leading to inhibition of the LH surge and then inducing the formation of a large follicular functional cyst

Theoretically, the anti-estrogenic effects on the hypothalamic-pituitary-axis also interfere with positive feedback, leading to inhibition of the LH surge (Fig. 2). Once the LH surge occurs, granulosa cells in the mature follicles will undergo luteinization, in which the steroid hormone production shifts from estradiol to progesterone (Devoto et al. 2002). Therefore, the formation of the single functional follicular cyst that produced estradiol at more than 500 pg/mL in the serum, strongly suggests the absence of the LH surge.

Although the direct effects of TAM on steroid hormone production of granulosa cells in the follicles were proposed to explain the high concentration of serum estradiol in the TAM-treated patients (Groom and Griffiths 1976), our findings could not confirm the presence of this mechanism since the inhibitory effects of TAM on estrogen-induced negative and positive feedbacks to the hypothalamic-pituitary-axis could successfully explain the ovarian hyper-stimulated phenomenon observed in this study.

It should also be noted that four cases, with a mean of 36.5 ± 8.35 years old, had been diagnosed with chemotherapy-induced menopause. However, a high value of serum estradiol was observed during the amenorrheic period that had continued from chemotherapy. Furthermore, in three of the four cases, these episodes were detected more than 1 year after the initiation of TAM treatment. From these findings, we suggest an important consideration that anovulatory hyperestrogenic conditions may be induced by TAM treatment even during the amenorrheic period in patients diagnosed with chemotherapy-induced menopause.

In addition, there was no significant difference in the duration from the start of single administration of TAM to the initial detection of a high concentration of estradiol between both chemotherapy-treated and non-treated groups. Importantly, although previous reports suggested that the development of an ovarian cyst is extremely rare after 2 years of TAM treatment (Madeddu et al. 2014), the average duration in both groups in this study was around 700 days, indicating that ovarian hyperstimulation can occur after 2-year treatment with TAM in Japanese women. Therefore, the risk of TAM-induced ovarian hyperstimulation should be considered throughout TAM treatment in Japan.

In conclusion, our findings indicate that anti-estrogenic hormonal treatment by TAM could stimulate the ovarian function even after single treatment for more than 2 years. Since single and multiple follicular developments were equally observed and follicular sizes were relatively large, we propose dual mechanisms of adverse effects of TAM on the ovarian function, that is, mediated through the inhibition of both negative and positive feedbacks to the hypothalamic-pituitary-axis.
